# Supplementary material for: Parkinson’s disease is characterized by vitamin B6-dependent inflammatory kynurenine pathway dysfunction
Source: NPJ Parkinsons Dis. 2025 Apr 26;11:96. doi: 10.1038/s41531-025-00964-7 (PMC12033312; doi:10.1038/s41531-025-00964-7)
Supplement: Supplementary file 1 — Supplementary Material [file 41531_2025_964_MOESM1_ESM.pdf]

Parkinson's disease is characterized by vitamin B6-dependent inflammatory kynurenine pathway dysfunction

Edward N. Wilson[1,2,3,*], Jacob Umans[1], Michelle S. Swarovski[1], Paras S. Minhas[1], Justin H. Mendiola[1], Øivind Midttun[4], Arve Ulvik[4], Marian Shahid-Besanti[1], Patricia Linortner[1], Siddhita D. Mhatre[1], Qian Wang[1], Divya Channappa[1,5], Nicole K. Corso[1], Lu Tian[6], Carolyn A. Fredericks[7], Geoffrey A. Kerchner[8], Edward D. Plowey[5], Brenna Cholerton[1], Per M. Ueland[4], Cyrus P. Zabetian[9,10], Nora E. Gray[11], Joseph F. Quinn[11,12], Thomas J. Montine[5], Sharon J. Sha[1,2], Frank M. Longo[1,2], David A. Wolk[13], Alice Chen-Plotkin[13], Victor W. Henderson[1,2], Tony Wyss-Coray[1,2,3], Anthony D. Wagner[14,2], Elizabeth C. Mormino[1,2], Nima Aghaeepour[6,15,16,17], Kathleen L. Poston[1,2,3,18] & Katrin I. Andreasson[1,2,3,19,*]

[1]Neurology & Neurological Sciences, Stanford University, Stanford, CA, 94305, USA
[2]Wu Tsai Neurosciences Institute, Stanford University, Stanford, CA, USA
[3]The Phil & Penny Knight Initiative for Brain Resilience, Stanford University, Stanford, CA, USA
[4]Bevital, Bergen, Norway
[5]Pathology, Stanford University, Stanford, CA, USA
[6]Biomedical Data Science and Statistics, Stanford University, Stanford, CA, USA
[7]Neurology, Yale University, New Haven, CT, USA
[8]Pharma Research and Early Development, F. Hoffmann-La Roche, Ltd., Basel, Switzerland
[9]VA Puget Sound Health Care System, Seattle, WA
[10]Neurology, University of Washington, Seattle, WA
[11]Neurology, Oregon Health & Sciences University, Portland, OR
[12]Neurology, Portland VA Medical Center, Portland, OR
[13]Neurology, University of Pennsylvania, Philadelphia, PA
[14]Psychology, Stanford University, Stanford, CA, USA
[15]Anesthesiology, Perioperative and Pain Medicine, Stanford University, Stanford, CA, 94305, USA
[16]Neonatal & Developmental Medicine, Department of Pediatrics, Stanford University, Stanford, CA, 94305, USA
[17]Biomedical Informatics, Stanford University, Stanford, CA, 94305, USA
[18]Neurosurgery, Stanford University, Stanford, CA, 94305, USA
[19]Chan Zuckerberg Biohub, San Francisco, CA 94158

*Correspondence should be addressed to: enwilson@stanford.edu & kandreas@stanford.edu

Supplementary Table 1. Association between KP metabolites and LEDD

	**Beta**	***P*-value**
**CSF**		
Tryptophan	-0.0472414	0.593
Kynurenine	0.09099964	0.293
Anthranilic Acid	0.06407181	0.456
Kynurenic Acid	-0.0534701	0.535
3-Hydroxykynurenine	0.1451412	0.132
3-Hydroxyanthranilic Acid	0.15319694	0.107
Quinolinic Acid	**0.1918272**	**0.025**
Nicotinamide	-0.1382005	0.588
N1-methylnicotinamide	0.09141035	0.340
**Plasma**		
Tryptophan	**-0.23108662**	**0.010**
Kynurenine	-0.07602555	0.395
Anthranilic Acid	0.1100432	0.207
Kynurenic Acid	-0.1034907	0.248
3-Hydroxykynurenine	0.03012308	0.651
3-Hydroxyanthranilic Acid	**0.20838716**	**0.012**
Quinolinic Acid	-0.02001114	0.821
Nicotinamide	0.16025579	0.084
N1-methylnicotinamide	0.1540876	0.100

Supplementary Table 2. Association between B-vitamins and LEDD

	**Beta**	***P*-value**
**CSF**		
Thiamine	-0.078	0.399
Thiamine Monophosphate	0.155	0.092
Riboflavin	-0.006	0.953
Pyridoxal 5'-phosphate	-0.170	0.071
Pyridoxal	-0.166	0.077
Pyridoxic Acid	-0.140	0.138
**Plasma**		
Thiamine	0.038	0.690
Thiamine Monophosphate	0.100	0.296
Riboflavin	0.149	0.119
Pyridoxal 5'-phosphate	-0.080	0.405
Pyridoxal	-0.165	0.096
Pyridoxic Acid	-0.017	0.850
4-PA	**-0.262**	**0.006**
Flavin Mononucleotide	0.089	0.334

Supplementary Table 3. Associations between KP metabolites and B-vitamins with age and sex

	Age		Sex	
**CSF**	**Beta**	***P*-value**	**Beta**	***P*-value**
**Tryptophan**	**0.012**	**0.143**	0.087	-0.097
**Kynurenine**	**0.001**	**0.219**	**0.043**	**-0.113**
**Kynurenic Acid**	**0.001**	**0.225**	0.670	0.023
**Anthranilic Acid**	**0.001**	**0.285**	0.622	-0.027
**3-Hydroxykynurenine**	**0.032**	**0.129**	0.336	-0.058
**Quinolinic Acid**	**0.001**	**0.341**	**0.013**	**-0.134**
**Nicotinamide**	0.717	-0.021	**0.005**	**-0.168**
**N1-Methylnicotinamide**	0.251	-0.068	0.381	-0.052
**Thiamine**	0.545	-0.035	0.121	0.090
**Thiamine Monophosphate**	0.989	0.001	0.496	-0.039
**Riboflavin**	0.120	-0.090	0.563	-0.033
**Flavin Mononucleotide**	0.799	0.014	0.052	-0.110
**Pyridoxal 5'-Phosphate**	0.588	-0.032	0.949	0.004
**Pyridoxal**	0.684	0.024	0.075	0.104
**Plasma**				
**Tryptophan**	0.418	-0.049	0.223	-0.074
**Kynurenine**	**0.010**	**0.154**	**0.011**	**-0.152**
**Kynurenic Acid**	0.069	0.110	**0.030**	**-0.132**
**Anthranilic Acid**	**0.001**	**0.287**	0.129	-0.089
**3-Hydroxykynurenine**	**0.015**	**0.155**	0.133	-0.095
**Quinolinic Acid**	**0.001**	**0.265**	0.181	-0.079
**Xanthurenic Acid**	0.747	-0.019	**0.001**	**-0.252**
**Nicotinamide**	0.791	-0.016	0.105	0.101
**N1-Methylnicotinamide**	0.972	-0.002	0.560	0.036
**Thiamine**	0.453	-0.048	0.606	0.033
**Thiamine Monophosphate**	0.793	0.016	0.141	0.091
**Riboflavin**	0.816	-0.015	0.779	-0.018
**Flavin Mononucleotide**	0.519	-0.039	0.122	0.094
**Pyridoxal 5'-Phosphate**	0.911	-0.007	0.394	0.053
**Pyridoxal**	0.941	0.005	0.778	-0.018

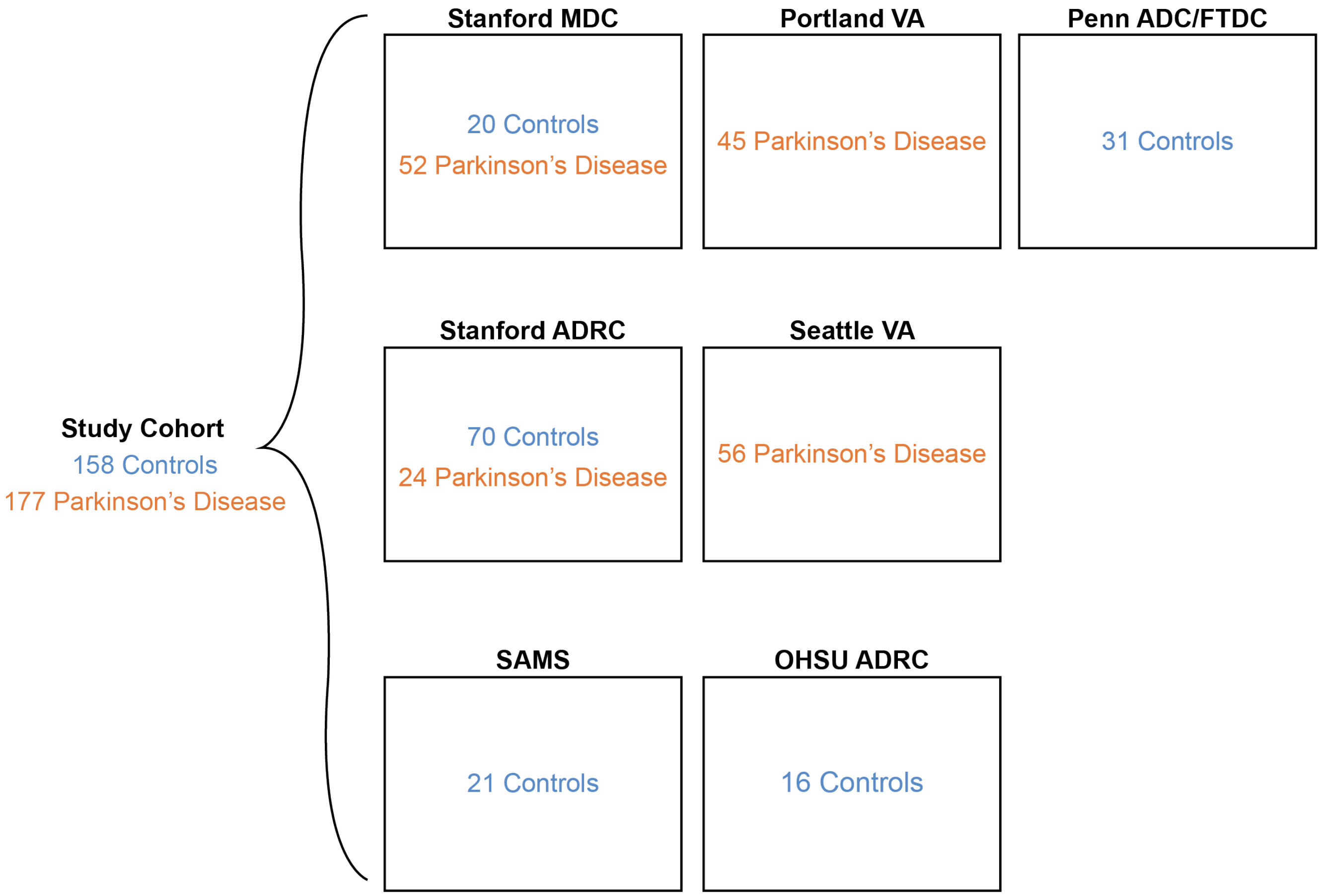

Supplementary Figure 1: Multicenter study of control participants and Parkinson's disease participants.

The combined study cohort consisted of a total of 158 control and 177 Parkinson's disease participants. Number of participants and diagnostic group are indicated for each recruiting research center. This multicenter study included participants from 1) the Stanford Movement Disorders Clinic (Stanford MDC); 2) the Stanford Alzheimer's Disease Research Center (Stanford ADRC); 3) the Stanford Aging and Memory Study (SAMS); 4) the VA Puget Sound Health Care System/University of Washington (Seattle VA); 5) the VA Portland Medical Center (Portland VA); 6) the Oregon Health & Science University Layton Aging and Alzheimer's Disease Research Center (OHSU ADRC); 7) the University of Pennsylvania Alzheimer's Disease Center (Penn ADC); and 8) the University of Pennsylvania Frontal Temporal Dementia Center (Penn FTDC). Demographic information on the study participants is included in **Table 1**.

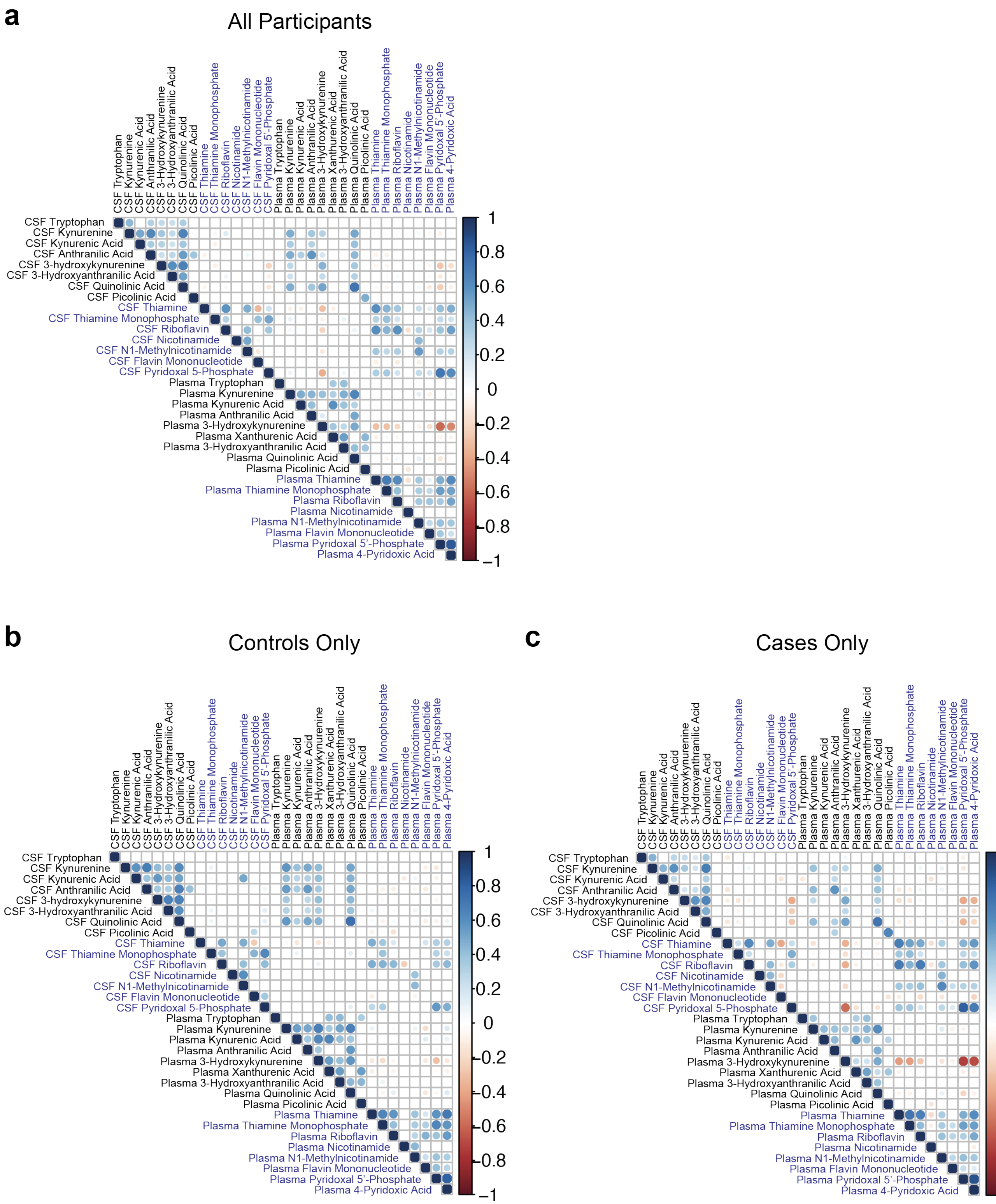

Supplementary Figure 2: Correlation Between CSF and Plasma Metabolic Pools.

Correlation matrix showing associations between CSF and plasma metabolites (black text) and related B-vitamins (blue text) in all participants (**a**), control participants only (**b**) and PD cases only (**c**). Significant correlations are shown with the circle size indicating the *P*-value of the correlation and color indicating size and direction of the *β*-estimate.

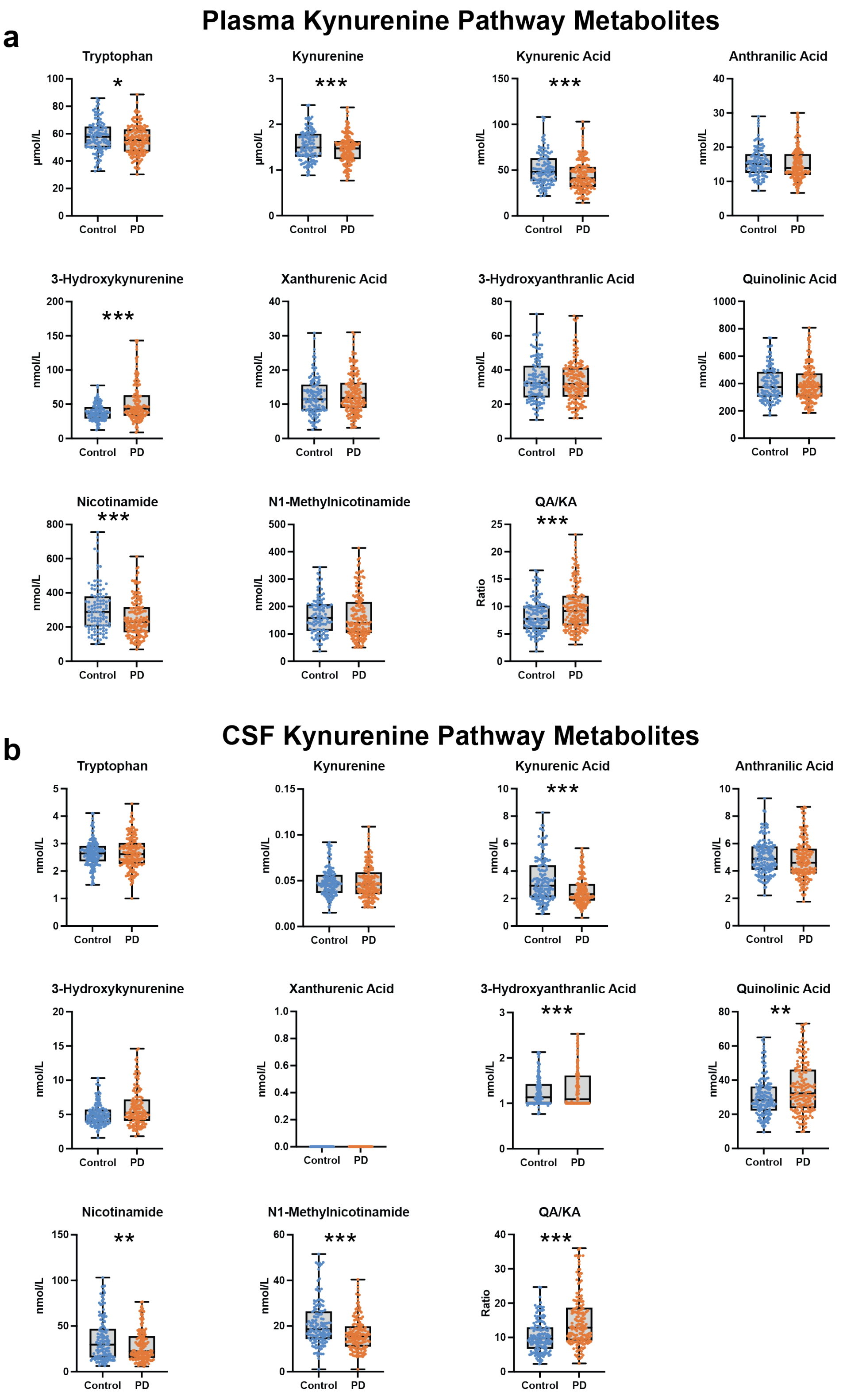

Supplementary Figure 3: KP changes in plasma and CSF metabolite pools in Parkinson's disease.

Concentrations of plasma (**a**) and CSF (**b**) kynurenine pathway metabolites in control and PD participants. **P* < 0.05 and ****P* < 0.001 using ANCOVA including age and sex as covariates.

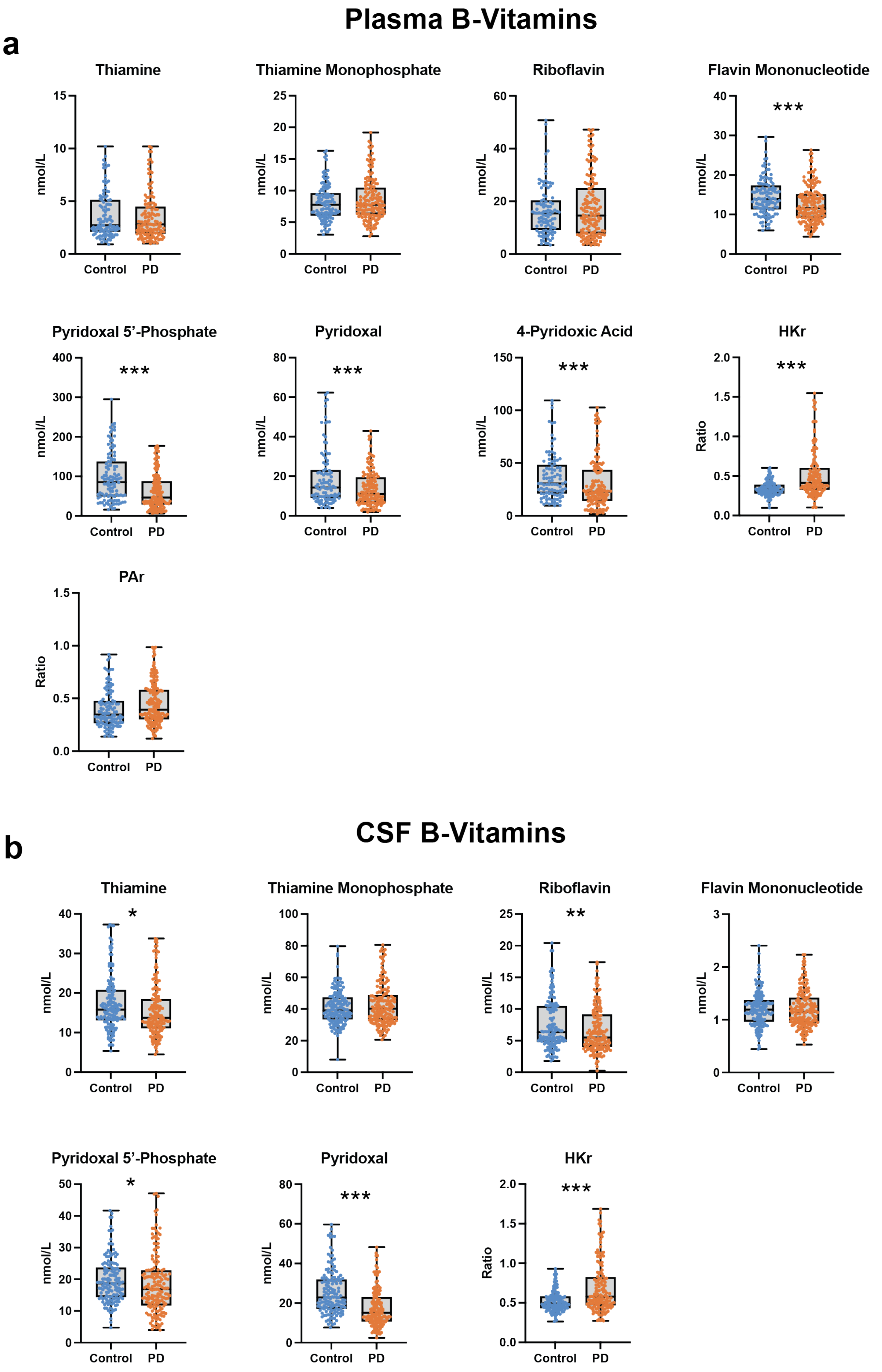

Supplementary Figure 4: Vitamin B deficiency in plasma and CSF pools in Parkinson’s disease.

Concentrations of plasma (**a**) and CSF (**b**) B-vitamins in control and PD participants. **P* < 0.05 and ****P* < 0.001 using ANCOVA including age and sex as covariates. Abbreviations: HKr: HK ratio; PAr: PAr Index.

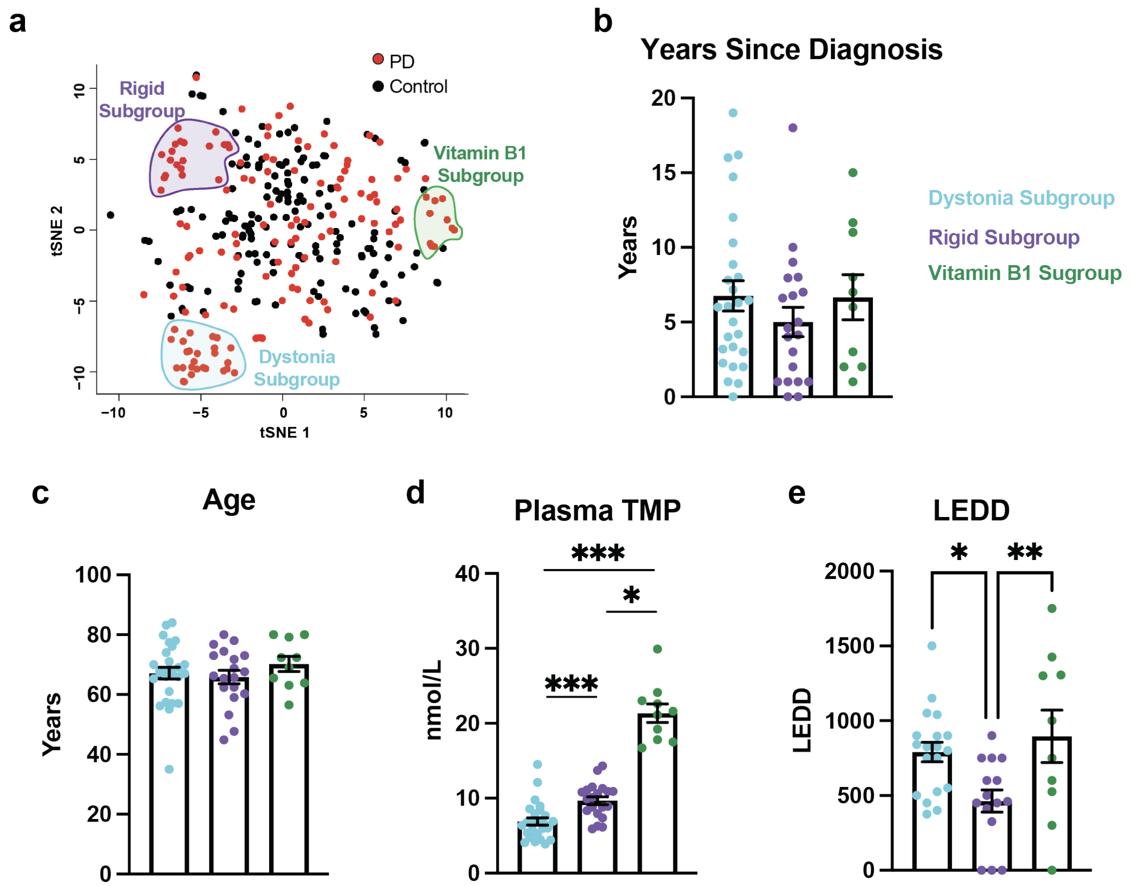

Supplementary Figure 5: Features of the PD clinical subgroups

(a) tSNE clustering of the natural classes of the entire study population by blood and CSF metabolomic data uncovered three subgroups of PD participants. Control participants: black circles. PD participants: red circles. Three distinct clusters of PD participants are circled. Dystonia Subgroup: cyan, Rigid Subgroup: purple, Vitamin B1 Subgroup: green.

(b) Mean years since diagnosis across the PD clinical subgroups. Group differences were assessed using one-way ANOVA with Tukey's post hoc test for pairwise comparisons.

(c) Mean age (years) across the PD clinical subgroups. Group differences were assessed using one-way ANOVA with Tukey's post hoc test for pairwise comparisons.

(d) Plasma thiamine monophosphate (TMP) across the PD clinical subgroups. Group differences were assessed using one-way ANOVA with Tukey's post hoc test for pairwise comparisons. **P* < 0.05, ****P* < 0.001.

(e) Mean levodopa equivalent daily dose (LEDD) in PD participants according to subgroups. Group differences were assessed using one-way ANOVA with Tukey's post hoc test for pairwise comparisons. **P* < 0.05, ***P* < 0.01.
